# 60 kD Ro and nRNP A Frequently Initiate Human Lupus Autoimmunity

**DOI:** 10.1371/journal.pone.0009599

**Published:** 2010-03-10

**Authors:** Latisha D. Heinlen, Micah T. McClain, Lauren L. Ritterhouse, Benjamin F. Bruner, Colin C. Edgerton, Michael P. Keith, Judith A. James, John B. Harley

**Affiliations:** 1 Department of Clinical Immunology, Oklahoma Medical Research Foundation, Oklahoma City, Oklahoma, United States of America; 2 Department of Internal Medicine, University of Oklahoma Health Sciences Center, Oklahoma City, Oklahoma, United States of America; 3 Department of Pathology, University of Oklahoma Health Sciences Center, Oklahoma City, Oklahoma, United States of America; 4 Walter Reed Army Medical Center, Washington, District of Columbia, United States of America; 5 National Naval Medical Center, Bethesda, Maryland, United States of America; 6 United States Department of Veterans Affairs, Oklahoma City, Oklahoma, United States of America; 7 Department of Arthritis and Immunology, Oklahoma Medical Research Foundation, Oklahoma City, Oklahoma, United States of America; New York University, United States of America

## Abstract

Systemic lupus erythematosus (SLE) is a clinically heterogeneous, humoral autoimmune disorder. The unifying feature among SLE patients is the production of large quantities of autoantibodies. Serum samples from 129 patients collected before the onset of SLE and while in the United States military were evaluated for early pre-clinical serologic events. The first available positive serum sample frequently already contained multiple autoantibody specificities (65%). However, in 34 SLE patients the earliest pre-clinical serum sample positive for any detectable common autoantibody bound only a single autoantigen, most commonly 60 kD Ro (29%), nRNP A (24%), anti-phospholipids (18%) or rheumatoid factor (15%). We identified several recurrent patterns of autoantibody onset using these pre-diagnostic samples. In the serum samples available, anti-nRNP A appeared before or simultaneously with anti-nRNP 70 K in 96% of the patients who had both autoantibodies at diagnosis. Anti-60 kD Ro antibodies appeared before or simultaneously with anti-La (98%) or anti-52 kD Ro (95%). The autoantibody response in SLE patients begins simply, often binding a single specific autoantigen years before disease onset, followed by epitope spreading to additional autoantigenic specificities that are accrued in recurring patterns.

## Introduction

High concentrations of autoantibodies are found in sera from nearly all patients with systemic lupus erythematosus (SLE), a heterogenous autoimmune disorder, and are important in many SLE clinical sequelae [1], [2]. These autoantibodies are frequently directed against dsDNA with associated nucleosome components, as well as common RNA-proteins such as Sm, nRNP, Ro, and La. Anti-dsDNA antibodies are found in about 50% of the sera from untreated lupus patients and are sufficiently specific for lupus that they contribute to the accepted SLE classification criteria [3], [4]. These antibodies are often associated with lupus renal disease [5]–[7]. Recent work has suggested that treatment of lupus with corticosteroids upon detection of rising dsDNA antibody titers and complement split products can oftentimes avert more serious clinical involvement [8]. Chromatin, histones and other nucleosome components are also commonly targeted by autoantibodies in SLE patient sera [9]. Approximately 25% of SLE patients produce antibodies against the Sm proteins of the spliceosome, particularly autoantibodies to the B/B' proteins [10], [11]. The related anti-nRNP antibodies, directed against nRNP 70 K, nRNP A, and nRNP C, are more prevalent but less specific for SLE [12]. Antibodies against the Ro autoantigen are present in approximately 50% sera from SLE patients [13], though even less specific, and generally bind a 60 kD Ro protein with many also binding a 52 kD Ro moiety.

Recent data suggest that lupus autoantibodies do not arise simultaneously, but rather develop sequentially over time [14], [15]. If true, then the first SLE specific autoantibody specificity establishes lupus humoral autoimmunity and may well be the conduit through which the formation of all subsequent lupus-related autoantibodies are generated, thus making the identification of the first lupus autoantigen bound critical to understanding lupus immune pathogenesis. Once initiated, epitope spreading provides a mechanism for the development of autoimmunity in SLE patients [16]–[22]. The anti-Sm autoantibody system, for example, progresses from a single initial epitope to a complex mix of multiple specificities revealing an active autoimmune developmental process [16]–. Similarly, the anti-60 kD Ro response begins from a single epitope and develops into a complex multi-epitope response [20]–[22].

While much effort has focused on identifying pathogenic mechanisms of these autoimmune responses, the early events in human SLE pathogenesis remain poorly understood. Patients are often diagnosed months after clinical illness onset and years after autoantibody production has commenced. Data are consequently sparse from the pre-diagnostic interval in human SLE development [14], [23]–[26]. Prospective serum collections such as the U.S. Department of Defense Serum Repository (DoDSR) provide access to serum samples which contain the cumulative immune histories of subsequent SLE patients, thus offering a unique opportunity to evaluate the immune system before clinical illness onset [14], [23]–[26]. We have found that autoantibodies consistently appear years before diagnosis of SLE within this cohort of patients [14]; however, we have not previously defined the historical order of protein-specific autoantibody appearance with the purpose being to identify the autoantibodies that first bind lupus autoantigens.

Thus, we sought to identify the first disease-associated autoantibody specificities that initiate autoimmunity in the process that culminates in SLE as a clinical illness. The earliest autoantibodies that develop in patients destined to develop SLE should be involved in the transition from normal immune regulation to autoimmune dysregulation and are therefore critically important components of the mechanisms of lupus pathogenesis. We show that the set of initiating autoantibodies is usually limited to Ro, nRNP A, phospholipids and rheumatoid factor of the specificities tested, suggesting that there appears to be an autoantigentic bottleneck that restricts autoantibody initiation to a relatively small number of initial antigenic structures on the pathway to SLE development.

## Results

### Prevalence and Time of Appearance of Autoantibodies before Diagnosis

Initial solid-phase autoantibody testing was performed by a commercial assay (Bio-Rad BioPlex ANA 2200, Hercules, CA) on 600 serum samples from 129 SLE patients and another 199 serum samples from 129 matched controls. These data were supplemented by Western blot analysis of 434 sera from 118 of the SLE patients. All of the results are from, and restricted to, sera within the normal range of Factor XIIIb.

**Figure 1:**
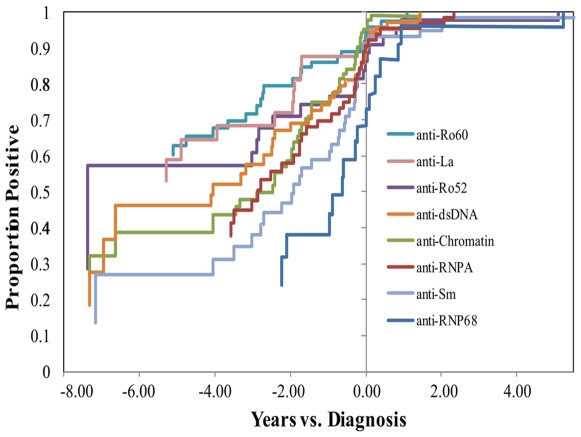
Autoantibodies precede lupus classification and occur in linked subsets. Kaplan-Maier survival curves for the onset of each autoantibody specific as measured by a solid phase, bead-based assay are presented. Anti-60 kD Ro, anti-La and anti-52 kD Ro are among the earliest specificities detected by this method. In contrast, anti-68 kD nRNP and nRNP A specificities are frequently detected closer to the time of lupus classification.

Of the 129 patients, 114 (88%) had detectable autoantibodies before SLE diagnosis. The most common antigen recognized before diagnosis was 60 kD Ro; 63 (49%) bound 601 kD Ro before 4 SLE criteria were present (Table 1). Antibodies to 60 kD Ro appeared an average of 3.54 years (median 3.12) before diagnosis, which was one of the earliest of specificities to appear relative to the time of SLE diagnosis (Figure 1). Anti-centromere B developed early but was only found in 5 (<5%) of the SLE patients and were usually only present transiently. Anti-60 kD Ro antibodies were more common in African-American patients than in European-American patients in this Military population (OR = 5.1; p = 0.0007) (Table 1).

**Table 1:** Prevalence and time of onset of autoantibodies as detected by the BioPlex 2200 ANA Screen kit and demographics of patients positive for each autoantibody.

	Number Positive		Time of Onset					
	Ever	Before	Mean	Median				
		Diagnosis			% female	% male	%EA	%AA
	% (n)	% (n)	Yrs prior to diagnosis		(n = 84)	(n = 45)	(n = 34)	(n = 80)
60 kD Ro	56 (72)	48 (63)	3.54	3.12	58	51	29	66*
La	36 (47)	30 (39)	3.05	2.42	37	36	24	41
52 kD Ro	36 (46)	29 (38)	2.49	2.26	42	24	26	43
nRNP A	50 (65)	42 (54)	1.85	1.71	52	47	32	63*
P	30 (39)	26 (33)	1.61	0.67	27	36	15	38
Sm	51 (66)	44 (57)	1.54	0.94	48	58	32	61*
dsDNA	42 (54)	33 (42)	1.26	0.53	40	44	32	45
chromatin	57 (73)	47 (61)	1.24	1.61	54	62	47	63
nRNP 70 K	19 (24)	14 (18)	0.68	0.61	21	13	21	20

Antibodies to nRNP A, Sm, and 60 kD Ro are significantly more common in African-Americans.

EA =  European-Americans.

AA = African-Americans.

*p<0.008.

Before diagnosis the prevalence of antibodies to chromatin (47%), Sm (44%), and nRNP A (42%) were similar to anti-60 kD Ro (49%). The first appearance of these specificities was more proximal to diagnosis with the mean onset of anti-chromatin (1.24 years before diagnosis), anti-Sm (1.54 years) and anti-nRNP A (1.85 years), occurring approximately half as close to classification as the average interval for anti-60 kD Ro (Figure 1). Anti-Sm and anti-nRNP A were also significantly more common in African-American patient sera relative to European-Americans (OR = 3.3 and OR = 3.5 respectively; p<0.008) (Table 1), supporting previously published studies [14], [15].

Twelve controls had autoantibodies detected using the commercial bead-based, solid phase assay. One control had antibodies to nRNP A, nRNP 70 K and chromatin that were present in both early and late samples. Sm, nRNP A, and centromere B were each bound by one control and these autoantibodies were detected in both an early and a late sample. Three controls had antibodies against dsDNA, two controls had antibodies against La, and one control had antibodies against ribosomal P; however, all six of these controls had an additional subsequent sample tested that was negative.

Prior to diagnosis, autoantibodies were detected by at least one of the commercial bead-based, solid phase assays in 88% of patients. Meanwhile anti-nuclear (ANA) immunofluorescent assays also detected autoantibodies in an overlapping but not identical 88% of these same pre-diagnostic SLE samples. Indeed, only 6 individuals were negative for ANA by both methods. Interestingly, in patients with a sample within the two years prior to diagnosis (n = 105), 86% had detectable levels of autoantibodies using the commercial bead-based assays, while 82% had autoantibodies detected using ANA immunofluorescence.

### The Earliest Autoantigen Target Was Identified for a Subset of Patients

Using the solid phase assay, we had 37 patients with only one detectable autoantibody specificity in their first available antibody positive sample; however, 3 patients had only anti-La detected but these 3 patients were additionally positive for anti-60 kD Ro by Western blot analysis. Thus, these 3 patients were considered to have multiple specificities in their first available sample and were included in the multiple specificity group for analysis. The remaining 34 patients had no additional specificities identified by Western blot analysis in the serum sample positive by solid phase assay.

**Figure 2:**
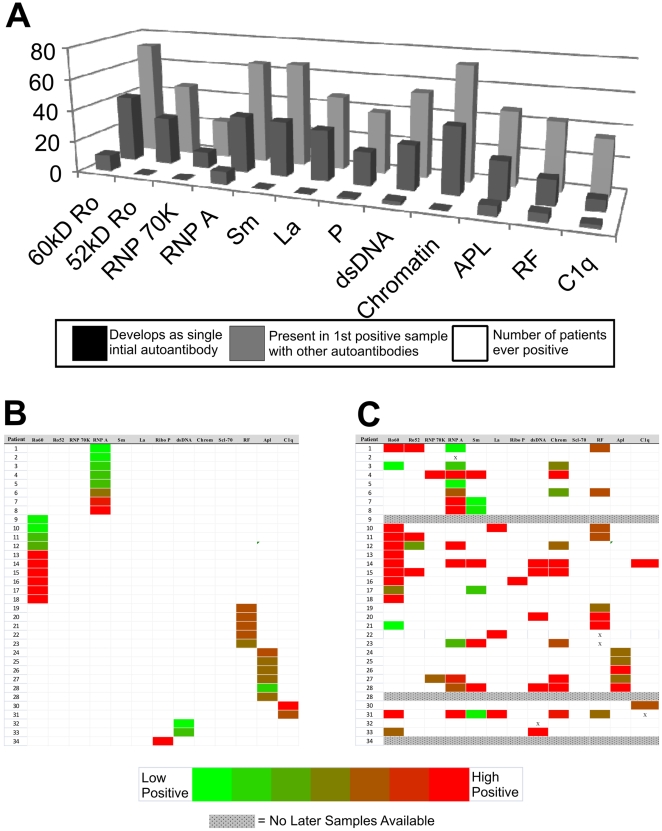
Initial autoantibody targets in the pre-diagnosis cohort. (A) The front (black) bars show the number of patients developing antibodies to respective antigen as their initial single autoantibody specificity. The middle (dark gray) bars show the number of patients with antibodies to the respective antigen in their first positive sample, which includes individuals who had multiple autoantibody specificities in their earliest positive serum. The back (light gray) bars show the total number of patients ever positive for each specificity. (B) Initial autoantibody profiles detected in 34 patients with a single initial specificity and (C) the final autoantibody profile for the patients with a later sample available. Colorimetric changes represent autoantibody concentration, ranging from green showing lower reactivity to bright red for high binding.

Of the 34 patients with a single initial specificity, 10 (29%) had detectable antibodies against 60 kD Ro, 8 (24%) to nRNP A, 6 (17%) to phospholipids and 5 (15%) had rheumatoid factor (IgG and/or IgM) as the initial sole lupus autoantibody detected. Unexpectedly, no patient had anti-nRNP 70 K or anti-Sm as the first sole detectable autoantibody. Antibodies to ribosomal P was found in one individual and antibodies to either dsDNA or C1q were found in two individuals each (Figure 2A, 2B). The final autoantibody profile in these 34 patients with an initial single autoantibody shows the development of antibodies against additional specificities in the majority of patients (68%) (Figure 2C).

### Lupus Autoantibodies May Be Initially Absent in Prediagnostic SLE Sera

Using the antigen-specific responses measured by luminex bead based assays, we had 25 patients for which at least one serum sample was negative and subsequent serum samples were positive for lupus-specific autoantibodies in the commercial assay. Nineteen patients had multiple autoantibodies and six patients had a single autoantibody in their first reactive serum sample. Five of the 6 patients with one initial specificity proceeded to develop multiple autoantibodies before meeting SLE classification criteria; therefore, a total of 24 of these 25 patients had multiple autoantibodies present before diagnosis. The time between the last negative and first positive sample was 1.32 years (median = 1.26 years) in the patients who had one autoantibody and 2.9 years (median = 3.35 years) in the patients who had multiple autoantibodies (p = 0.027). No significant differences in gender, race, or age at diagnosis were found between the patients with a single initial autoantibody and those with multiple specificities.

The antigens targeted in the initial positive sample of the 25 patients who developed autoantibodies under observation are largely (89.5%) detectable in the subsequent autoantibody profile. Of the 129 SLE patients, 84 had multiple autoantibody specificities detectable in their first autoantibody positive serum sample. The early autoantibodies discovered by analysis of these individuals with negative samples followed by sole specificities were also among the specificities recognized by samples from individuals with multiple autoantibodies present in the first available sample.

### Accumulation of Antibodies within Linked Systems Follows Distinct Patterns

**Figure 3:**
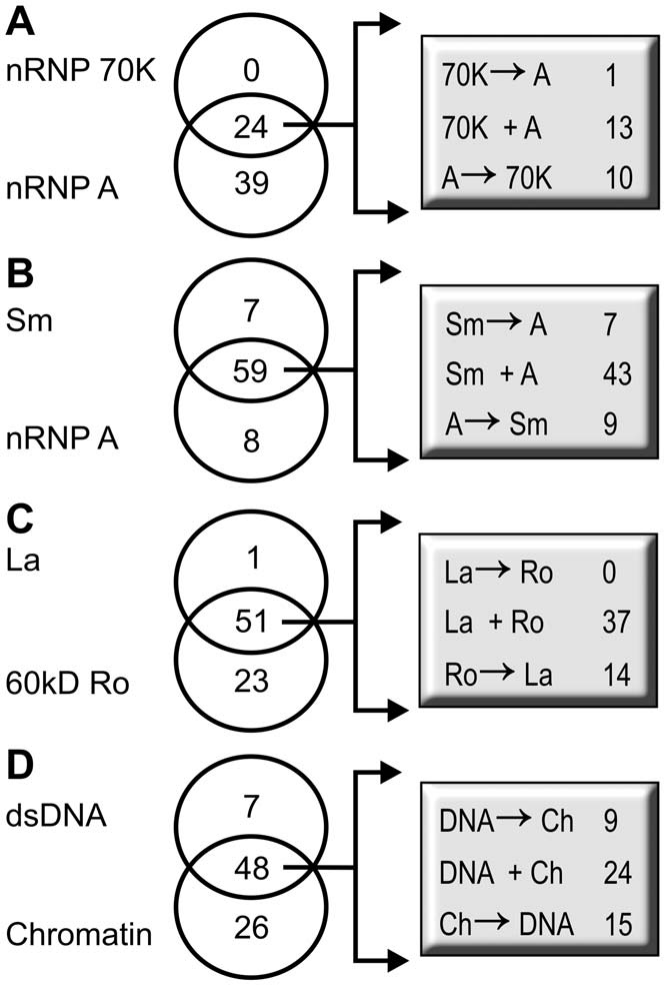
The order of onset of protein-specific autoantibodies within the linked systems. Antibodies to nRNP A commonly appeared prior to anti-70 K (A) while a variable onset was seen between Sm and nRNP A (B). Anti-60 kD antibodies virtually always precede anti-La antibodies (C). The order of appearance did not favor anti-dsDNA or anti-chromatin when compared to each other (D).

When analyzing all patients in this study, 63 had antibodies to nRNP A at some time during the pre-diagnostic period. Of these 63 patients, 24 also had antibodies to nRNP 70 K while the other 39 had no detectable anti-nRNP 70 K at any tested time point. Thirteen of the 24 patients had antibodies to both nRNP 70 K and nRNP A that were detected simultaneously, in the same serum sample. Antibodies to nRNP A preceded antibodies to nRNP 70 K in 10 patients, while antibodies to nRNP 70 K preceded antibodies to nRNP A in only 1 patient (Figure 3A). When all available cases are considered, 11 cases allow us to discriminate between the appearance of anti-nRNP A from anti-nRNP 70 K (Figure 3A) with anti-nRNP A appearing before anti-70 K in 10 and the reverse in only one of the 11 individuals with informative sera (OR = 10, p = 0.002). This observation is consistent with the possibility of a sampling issue in a substantial proportion of the 19 with multiple specificities in the earliest positive serum. Under this scenario a serum sample would not have been collected during the time that a single initiating autoantibody would have been present.

A subset of these samples was studied by Western blotting against HeLa cell extract. The samples selected had anti-nRNP 70 K and anti-nRNP A first together (17 samples in the solid phase assays) along with those in which anti-nRNP A was detected first alone (25 samples by solid phase assay). Western blots confirmed the findings that no patient had anti-nRNP 70 K antibodies as an initiating autoantibody alone and that in no instance did anti-nRNP 70 K appear before anti-nRNP A by Western blot (data not presented).

Of the total collection tested, 59 patients had antibodies against Sm and nRNP A (detected by solid phase or Western blot) while 8 had anti-nRNP A without anti-Sm and 7 had anti-Sm without anti-nRNP A. Of the 59 patients who had both anti-Sm and anti-nRNP A, 43 had both specificities in the same sample. Antibodies to nRNP A appeared before antibodies to Sm in 9 patients and anti-Sm appeared before anti-nRNP A in 7 patients (X^2^ = 0.250, p = 0.617) (Figure 3B).

Antibodies against La were found in a subset of those with anti-60 kD Ro. Antibodies to both 60 kD Ro and La were found in 51 individuals. Anti-60 kD Ro and anti-La appeared simultaneously in 37 of these 51 patients (Figure 3C). In 14 of these 51 patients anti-60 kD Ro antibodies appeared prior to anti-La. In addition, anti-La antibodies did not appear before anti-60 kD Ro antibodies in any case that eventually developed both autoantibody specificities. Overall, we can discriminate the earlier appearance of anti-60 kD Ro in 14 patients while anti-La did not appear earlier in patients developing both specificities (OR  = 14.0, p = 0.0002) (Figure 3C).

Antibodies to both dsDNA and chromatin were present in 48 patients; anti-chromatin without anti-dsDNA was present in 26 patients and anti-dsDNA without anti-chromatin was seen in only 7 patients (Figure 3D). Of the 48 patients who had both antibodies present, 15 had anti-chromatin preceding anti-dsDNA, while 9 had anti-dsDNA preceding anti-chromatin. Twenty-four patients had antibodies to dsDNA and chromatin that appeared in the same serum sample. Overall, 15 patients had anti-chromatin first and 9 patients had anti-dsDNA first (X^2^ = 1.5, p = 0.22) (Figure 3D).

### Appearance of Autoantiboides Detected by a Bead-Based Assay Compared to Traditional Assays

The sera used in this study have previously been tested for autoantibodies using the traditional assays used by clinical laboratories, as described in previous publications [14], [23]–[26]. The data obtained using this solid-phase, bead-based assay were compared to these assays for autoantibodies time of appearance.In this collection there were 69 cases where the sera available discriminate between the earlier appearance of a positive antinuclear antibody (ANA) or a lupus-specific autoantibody. In 22 cases, the immunofluorescent ANA was detected first, while in 47 a specific autoantibody appeared first. Antibodies to common lupus antigens as detected by this solid-phase, bead-based assays were detectable prior to antibodies against cardiolipin (APL), Rheumatoid Factor (RF), and C1q in the majority of patients in which both were present (n = 79, n = 98, and n = 85, respectively). However, some patients developed antibodies to APL (6 patients), RF (5 patients), and C1q (2 patients) before antibodies to the other autoantigens tested were detectable.

The ANA test results constitute a special situation where something on the order of 10,000 antigens are tested simultaneously, including the 12 specific autoantigens being reported herein. The ANA was positive along with one of the 12 specific autoantigens individually tested in 14 of the first positive serum from the 34 subjects with a single specificity. In two of these the ANA was positive in an earlier serum (from the five of these 34 who had an earlier serum available). This result suggests that some additional autoantigens may be important in lupus serological progression beyond those tested. While single autoimmunity initiating specificities cannot be concluded for these subjects, a positive ANA, without other specific autoantibodies detected, is not inconsistent with this possibility.

## Discussion

Lupus disease expression is derived from the consequences of humoral autoimmunity. We show that lupus-specific autoimmunity evolves from a limited set of autoantigens, with most of the initiating autoantibodies being anti-60 kD Ro, anti-nRNP A, aPL or RF, but unexpectedly apparently anti-Sm and anti-nRNP 70 K being excluded as specificities that initiate lupus autoimmunity alone. This conclusion is bounded by the time between the forming autoantibody against initating autoimmune specificity and the second specificity. Because sera collected from each subject were separated by months to years, it is possible that if a second specificity follows the first rapidly, by day to weeks, then we would detect dual specificities appearing together in the collection studied herein. This is a formal possibility for anti-nRNP A and anti-nRNP 70 K and for the anti-60 kD Ro and anti-La, among others, though parsimony and the data showing that autoantibodies can first form against nRNP A and 60 kD Ro argue against this possibility.

The initiating autoantibodies are, at the point first detected, the earliest evidence in the patient of a lupus-specific autoimmune process. That they are a small subset of the known lupus-specific autoantibodies suggest that the number of structures through which human lupus is initiated is very restricted. This is the lupus autoantigenic bottleneck through which the autoimmune process must past to culminate in clinical inflammatory illness. If the subsequent extraordinary complexity of lupus-specific humoral autoimmunity is dependent upon these relatively simple beginnings, then epitope spreading, as previously shown in animal models [16], [18], [20]–[22] and in man [17], [18], [20], is a critical process for lupus autoimmune development and maturation. At the moment there is no evidence supporting the alternative hypothesis that new lupus specificities arise de novo, independent of and uninfluenced by the lupus autoimmune specificities that precede them.

Indeed, epitope spreading appears to follow some limited pathways that are evident from the data available herein. Anti-60 kD Ro precedes anti-La and anti-52 kD Ro in those subjects who eventually develop two or three of these autoantibodies. Anti-nRNP A precedes anti-nRNP 70 kD and anti-nRNP C, following an analogous pattern of developing complexity. Meanwhile, the temporal relationships between anti-Sm and ant-nRNP A and between anti-dsDNA and anti-chromatin do not show a dominating temporal relationship (Figure 3).

The most extensive previous studies of autoantibody development were performed using sera from a large cohort of connective tissue disease patients. Concentrating on patients already diagnosed with a systemic autoimmune rheumatic disease (many with mixed connective tissue disease) Greidinger and Hoffman [15] found that the spliceosomal autoimmune responses frequently began by targeting the nRNP 70 K protein followed by Sm B'. Anti-nRNP A and anti-nRNP C developed later, while antibodies to Sm D1 developed as the last of all the common snRNPs. Ro, La, dsDNA, chromatin or ribosomal P autoantibodies were not evaluated [15]. The most obvious explanation for the differences between their identification of anti-nRNP 70 K as an initiating autoantibody and our identification of anti-nRNP A is that their collection was enriched for mixed connective tissue disease. Meanwhile, this report strictly concerns lupus. The initiating autoantigen could easily be a disease distinguishing feature in disease pathogenesis.

Existing evidence supports a model for anti-Ro (in those patients who do not also develop anti-La) [20] involving Epstein-Barr virus Nuclear Antigen-1 (EBNA-1) as the cross-reacting hetero-antigen that mediates the antibody cross-reaction making 60 kD Ro an initiating lupus autoantigen [20]. Anti-Sm B autoantibodies are initiated through PPPGMRPP [17], [18], [27], which is repeated exactly or nearly exactly four times in the carboxy-terminus of Sm B. But the results herein do not show that Sm is an initiating autoantigen for lupus. Both nRNP A with PPPGMIPP and nRNP C with PAPGMRPP have epitopes that cross-react with anti-PPPGMRPP autoantibodies [28]–[30].

**Figure 4:**
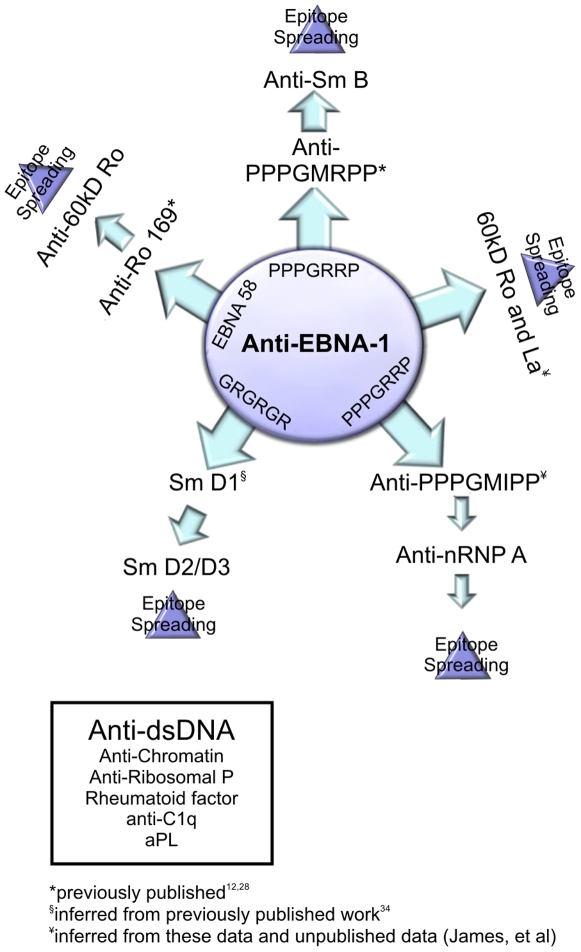
Potential mechanism for development of autoantibodies through a common environmental etiology. While potential structural mimics are known with EBNA-1 for Ro, Sm B, Sm D, nRNP A, nRNP C and nRNP 70 K, no such structural relationship is known for a heteroimmune response operating to generate anti-dsDNA, anti-chromatin or anti-ribosomal P.

EBNA-1 appears to be the primordial hetero-antigen with anti-PPPGRRP of EBNA-1 cross-reacting in a way that transforms the hetero-immune response into an autoimmune response [31], [32]. Other autoantigen specific molecular mimicry relationships between EBNA-1 and spliceosomal antigens have been proposed [33], [34]. Existing data are consistent with a model that starts with anti-EBNA-1 and then initially cross-reacts with anti-PPPGMIPP (Figure 4), but this progression, which is in preference to the anti-PPPGMRPP of anti-Sm, has not yet been formally tested.

This study is limited by the availability of stored serum samples and presumptions based upon the timing of the samples collected. Many of our critical observations are made from the 34 patients who are documented to begin with a single initial autoantibody specificity prior to clinical classification with lupus. Since our conclusions concerning the initiation of lupus autoimmunity are derived from a minority of the subjects considered, the potential distorting influence of ascertainment bias should be weighed. Furthermore, the use of standardized solid phase assays and Western blots allows for some variation in detection sensitivity and specificity between autoantigens and techniques, which is made more problematic by making many of these measurements as close to the limit of detection as is possible. We are reassured that the solid phase assay results presented herein closely resemble the proportions found in previous studies focusing on Western blot results in a different cohort [14]. We are also reassured by the high concordance between Western blot and the commercial solid phase bead assay results. The concordance of anti-60 kD Ro, anti-La, anti-Sm and anti-nRNP between the two assays was 90%, 80%, 85% and 84%, respectively, with none of the differences making a material difference to the interpretations offered; specifically, the rate and order of appearance of the autoantibodies tested by ELISA did not differ from the solid phase autoantibody data presented.

Despite the limitations and uncertainties that these constraints and considerations place on the interpretation of the results, the data are at a minimum consistent with a number of broad interpretations and, in the context of other work, contribute to a more detailed model of lupus immunopathogenesis as follows. Lupus begins as a less complicated autoimmune process, often involving a single lupus-specific reactivity. The single specificities that are able to initiate lupus autoimmunity include at least the autoimmune responses against 60 kD Ro, nRNP A, dsDNA, phospholipid, and ribosomal P. For lupus the initiating autoantogens do not generally include nRNP 70 K or Sm. The data are also consistent with anti-Ro always preceding anti-La and with anti-nRNP A preceding anti-nRNP 70 K. Anti-dsDNA as a first specificity precedes anti-chromatin, but later and in the presence of other specificities in the serum anti-chromatin may precede anti-dsDNA. All in all, these data are consistent with the possibility that lupus autoimmunity as detected by autoantibody binding is initiated by a single autoantibody specificity, which is also consistent with the progression of epitope spreading in lupus described previously [16]–[22] and, also, hereby becomes a hypothesis to be further tested by future experiments.

To a limited extent these data also address the relative disease process specificity of the initiation of lupus autoimmunity. The less lupus-specific autoantibodies directed against rheumatoid factor or just anti-nuclear antibodies, when also present at diagnosis in these cases, may or may not precede lupus-specific autoantobodies. That they do not in the majority of cases re-enforce the contention that at its fundamental level of autoimmune initiation lupus is an autoantigen-specific disorder and that many of the non-specific autoantibodies arise subsequently as a consequence of lupus immunopathogenesis.

In summary, the development of autoantibodies in some SLE patients appears to begin with a simple autoimmune response binding a single autoantigen. These proteins are members of a restricted set of autoantigens known to be capable of being the initiating target for human lupus autoimmunity. Previous work suggests that at least some of these antibodies arise from the anti-EBNA-1 heteroimmune response. After passing through this autoantigenic bottleneck, the initial humoral autoimmune response then usually diversifies, with the sequential development of autoantibodies binding multiple specificities before clinical presentation and diagnosis. Common patterns of autoantibody development suggest common underlying mechanisms within these subsets. Similar initial target epitopes suggest similar origins. Despite the impressive clinical and serologic diversity seen in SLE, we demonstrate a relatively limited and definable set of conserved humoral development profiles that initiate and are present before clinical disease arises. The essential elements for some human lupus patients would be a particular hetero-immune response (e.g., anti-EBNA-1) that generates heteroimmune and autotimmune cross-reacting antibodies (e.g, EBNA-1 and 60 kD Ro). From this small but dangerous beginning the immune response penetrates the autoantigenic bottleneck. The initiating autoantigen (e.g, 60 kD Ro) in lupus is a sufficiently robust auto-immunogen to sustain the expansion and increasing complexity of the autoimmune response culminating in this life-threatening autoimmune disease.

## Materials and Methods

### Study Population and Design

Appropriate IRB approval was obtained from the Oklahoma Medical Research Foundation, the University of Oklahoma Health Sciences Center, the Walter Reed Army Medical Center, and National Naval Medical Center. Use of coded, previously collected samples waived the necessity for informed consent. The research was conducted in compliance with the Helsinki Declaration (version 2000).

Military rheumatologists queried computer databases of military hospital records and other clinical records to identify individuals with a potential SLE diagnosis (based upon the ICD-9 code 710.0). Inclusion criteria included having been diagnosed with SLE while serving in the United States military or reserves, satisfying any 4 of the 11 American College of Rheumatology (ACR) SLE classification criteria [3, 4), having at least one retrievable serum sample that was collected before clinical SLE diagnosis and having a sufficient amount of sera available for this study from the Department of Defense Serum Repository (DoDSR) [14], [23]–[26].

Cumulative clinical features and laboratory results were collected from the chart of each identified SLE patient, particularly the date of the first appearance of each ACR clinical criterion. SLE diagnosis was determined by the military clinician (CCE). Demographic information included self-reported race/ethnicity, gender and age at disease onset, which was classified as meeting at least one ACR clinical criterion for SLE classification [3], [4]. Once all SLE patients were identified, the Department of Defense Serum databases were queried to identify SLE-unaffected control individuals. Four controls were matched for each patient based on self-reported race/ethnicity, gender, age, length of service in the military, and number of available samples in the repository. For this study, one matched control for each patient was randomly selected as a control for the autoantibody assays. Both the first and last available sample were analyzed from each control that had more than one sample available (n = 70).

### Autoantibody Analyses by Bead-Based, Solid Phase Assays

Undiluted serum samples from patients and controls were assayed using a commercially available bead-based, solid phase technology, the BioPlex ANA Screen™ kit performed with the BioPlex 2200™ instrument (Bio-Rad, Hercules, CA) [35]–[38]. These assays use multiplex technology and dyed magnetic beads to simultaneously perform measurements of 13 autoantibodies within 5 µl of an individual serum sample. This kit enabled the detection of the antibodies against any of the following antigens: SS-A 60 (60 kD Ro), SS-A 52 (52 kD Ro), SS-B (La), SmRNP complex, Sm, RNP 68, RNP A, centromere B, Scl-70 (topoisomerase 1), Jo-1, chromatin, dsDNA, and ribosomal P [35]–[38]. Centromere B, Jo-1, nRNP 70 K, nRNP A, Scl-70 (topoisomerase 1), and 52 kD Ro were all recombinantly produced; dsDNA was synthesized by polymerase chain reaction; and the remaining antigens were affinity purified from calf thymus extract by the manufacturer.

The BioPlex 2200™ system, a fully automated, random access analyzer, reports semi-quantitative values from 0–8, termed the antibody index (AI), for each autoantibody except anti-dsDNA. The positive cut-off for each assay was established by the manufacturer to equal 1.0 AI. For anti-dsDNA this method performs a quantitative assay; the results of which are reported as IU/mL with a positive threshold of 10 IU/mL. Guidelines from the manufacturer for the use of these control cut-offs were based upon use with control sets from a somewhat uniform, Caucasian-based healthy control population. Based upon the demographic nature of this cohort, including 62% African American, 10% Hispanic, 2% Asian and only 26% of European-American ancestry, we determine sample positivity based upon antibody binding of ≥4SD above the normal mean of a cohort of 130 healthy military individuals with the same racial, age and gender composition of our experimental lupus cohort. Internal quality control measures are used for assays. Positive, negative and calibrator controls were required to all measure within a standard range. Serum Factor XIIIb levels were tested in all samples to ensure quality control of the sera and to ensure sample integrity and appropriate testing. This method is a standard feature of the commercial assay and has been useful to determine the integrity of the samples and to determine whether the samples have been properly handled during long-term storage (data proprietary and not presented). Of the 600 SLE and 199 control samples that were tested, 67 and 5, respectively, had low Factor XIIIb errors indicating possible degradation. These sera were dropped from our analysis, which did not alter the interpretation of our results. Significantly more factor XIIIb errors were found in sera from patients than in sera from controls (OR = 4.9; p<0.001); however, no unifying characteristics were identified in the patient samples with low factor XIIIb levels. They were not samples from the same people, stored in the same boxes, nor collected during any specific time frame. Factor XIIIb levels in SLE patients have not been systematically studied to our knowledge.

### Western Blot Analysis

Electrophoresis was performed on HeLa cell extracts using 12.5% polyacrylamide gels under denaturing conditions and transferred to nitrocellulose. The nitrocellulose was then used for Western blotting, as previously described [18], [20], [39]. Autoantigens were determined to be present when two blinded observers identified distinct bands that corresponded to the appropriate molecular weight and to the band from the known positive control serum used in the same assay.

### Standard Autoantibody Assays by ELISAs and Immunofluorescence

All samples were tested for autoantibodies (IgM and/or IgG) directed against cardiolipin, C1q, and rheumatoid factor (RF), as previously described in detail [25], [26]. All samples were also tested for anti-nucelar antibodies by immunofluorescence on a HEp-2 cell substrate (ANA). All anti-dsDNA positive samples by the commercial bead-based, solid phase assay were also tested for anti-dsDNA by immunofluorescence against *Crithidia lucillae* as previously described [14], [23].

### Statistical Analyses

For the commercial assay results, the threshold for positive binding was set by using the control sera at mean binding plus four standard deviations. Control values for each of the 13 autoantibodies were normally distributed. For each antibody, mean times from first observed positive test to diagnosis were calculated using all individuals who ever developed that specificity. A Student's t-test was used to test for differences in the time of onset of different autoantibody specificities relative to the age at diagnosis or meeting a clinical criterion. Categorical variables (such as ethnicity and gender) were assessed by the chi-square statistic or Fisher's exact tests. Chi squares were used as appropriate to compare the order of autoantibody appearance.
